# Are we aiming to miss in translational autoimmunity treatments?

**DOI:** 10.12688/f1000research.16894.2

**Published:** 2019-01-08

**Authors:** Gisela M. Vaitaitis, David H. Wagner

**Affiliations:** 1 Webb-Waring Center, University of Colorado, Anschutz Medical Campus, Aurora, CO, 80045, USA; 2 Department of Medicine, University of Colorado, Anschutz Medical Campus, Aurora, CO, 80045, USA

**Keywords:** autoimmunity, treatment, translational, circadian, timing, dosing

## Abstract

Autoimmunity treatments, fruitfully pioneered in mouse models, can be disappointing or result in immunosuppression and opportunistic infections in translational trials. Many possible reasons exist, but one major, overlooked reason may be the treatment timing in relation to circadian oscillations of the immune system. Mice and humans both have immunological circadian clocks and experience the same circulatory oscillations of immune cells with regards to their sleep/wake phases, but have opposite sleep/wake phases with regard to the daylight cycle. Therefore, researchers mainly study mice and potential autoimmunity treatments during the murine sleep/rest phase, which is when pro-inflammatory mediators and more adaptive immune cells are prevalent in the circulation. In translational trials, however, treatment administration happens primarily during a patient’s wake/activity phase, during the daytime, which is when more local and acute immune responses are active in the circulation. Therefore, we believe that the most opportune window for autoimmunity treatment may be missed in translational trials. Shifting the timing, and adjusting dosing to target only immune cells that are active at that time, may result in higher success with minimized immunosuppression or toxicities.

## Translational treatments of autoimmunity often do not reflect findings in mouse models

Mouse models of autoimmunity are widely used and, while they are not perfect, they are often the best available tools for research in potential treatments for a number of human autoimmune diseases such as type 1 diabetes, rheumatoid arthritis, and multiple sclerosis (MS), to name but a few. In such mouse models, many potential therapeutics demonstrate great effects in preventing the development of disease, ameliorating the disease symptoms, or even curing the fulminant autoimmunity ^1– 5^. However, when applying therapeutics in translational medicine, clinical trials often are disappointing, demonstrating low or short-lived efficacy ^6– 8^. Many therapeutics come with toxicities that, in some cases, can be mitigated. However, once toxicities are addressed, the efficacy often remains at a point of ameliorating symptoms to some degree, but not in all patients, and rarely do the therapeutics cure the patients ^6, 9^. There are successful treatments that ameliorate human autoimmune disease; however, those treatments can be broadly immune suppressive, causing opportunistic infections in the patient ^10, 11^.

Differences in success of autoimmunity treatments in mice compared to humans could certainly be due to that autoimmunity in humans is different from the mouse models. The mouse models likely address only some of the possible etiologies of human autoimmune disease when the human disease consists of many different facets and etiologies, genetics, environmental differences, relative exposures etc., that converge on the same types of symptoms. Therefore, those etiologies are not all treatable with one strategy that may have been elucidated in the mouse model. However, when directing the autoimmunity therapy at events upon which all the etiologies may converge, such as killing or tolerizing T cells that are attacking self-tissues, why do therapies not have better outcomes? Could timing be at the core?

In this opinion paper, we wish to highlight one major overlooked possibility for some of the discrepancies between the findings in mouse and human autoimmunity treatments. We are by no means dismissing other possibilities since there are likely many reasons for the discrepancies; however, we feel that the particular possibility presented in this piece has yet to be considered.

## Circadian rhythm of the immune system and autoimmunity treatments

In 2017, the Nobel Prize in Physiology or Medicine was awarded for work on understanding circadian rhythm ^12^. That work has led to the understanding that not only is there a “master clock” that governs the biorhythm of humans and animals alike, but there are many organ specific “clocks” that turn individual organ processes on and off, perhaps several times in a 24 hour period. This turns out to be true for the immune system as well ^13^. Interestingly, there are different circadian rhythms for different parts of the immune system. Basal plasma levels of pro-inflammatory cytokines such as IL-1β, TNFα, IFNγ, and IL-6 are higher during the sleep/rest phase and are paralleled by an abundance of memory and naïve T cells in the circulation ^13^. Contrarily, anti-inflammatory cytokines, such as IL-4 and IL-10, increase upon awakening and CD8 ^+^ effector T cells as well as natural killer T cells peak during the wake/active phase ^13^. This results in more local cytotoxic activities during the active phase, when it is also more likely that wounding and acute pathogen and antigen exposure will occur ^13^.

The diurnal oscillation in T and B cells in the circulation is paralleled by an opposite oscillation phase in the lymph nodes ^14, 15^. These oscillations have implications in different disease courses as well as for vaccinations. For example, the disease course in the experimental autoimmune encephalomyelitis (EAE) mouse model of MS is significantly more severe if the disease induction regimen is given during the light cycle (when mice sleep/rest) than if given during the dark cycle (when mice are awake/active) ^14^. Similarly, the magnitude of Leishmania infection is dependent on the circadian time of infection ^16^. In humans, the timing of vaccination has been demonstrated to have an impact on the efficacy of the vaccine, where giving the influenza vaccine to patients in the morning enhanced the antibody response compared to when giving it in the afternoon ^17^. On the flip-side of this, severe disturbances in the sleep/wake cycle, such as during shift work, has been shown to cause significant health problems, including increased association with autoimmune disease ^18^. Given the circadian oscillations of the immune system and the impact of those oscillations on infection and vaccination/disease induction, it is not farfetched to consider that the efficacy of immune system targeting treatments would also be affected by the timing of treatment administration in relation to such oscillations.

While autoimmunity has many components contributing to the specific disease, it is known that CD4 T cells, also known as pathogenic effector cells, are the main drivers of many autoimmune diseases ^19– 22^. In addition, those driver T cells interact with, and activate, B cells to produce autoantibodies and/or present antigen, which further drives the autoimmune disease ^23, 24^. Different subsets of CD4 effector T cells and B cells are more or less prominent depending upon the particular autoimmune diseases ^21, 25– 27^. Those cells, being part of the adaptive immune system, are more prominent in the circulation during the sleep/rest phase in both mice and humans. Therefore, it would make sense to administer autoimmunity treatments that are directed at CD4 T cells and B cells at times when those cells are more prominent in the circulation.

**Figure 1.**
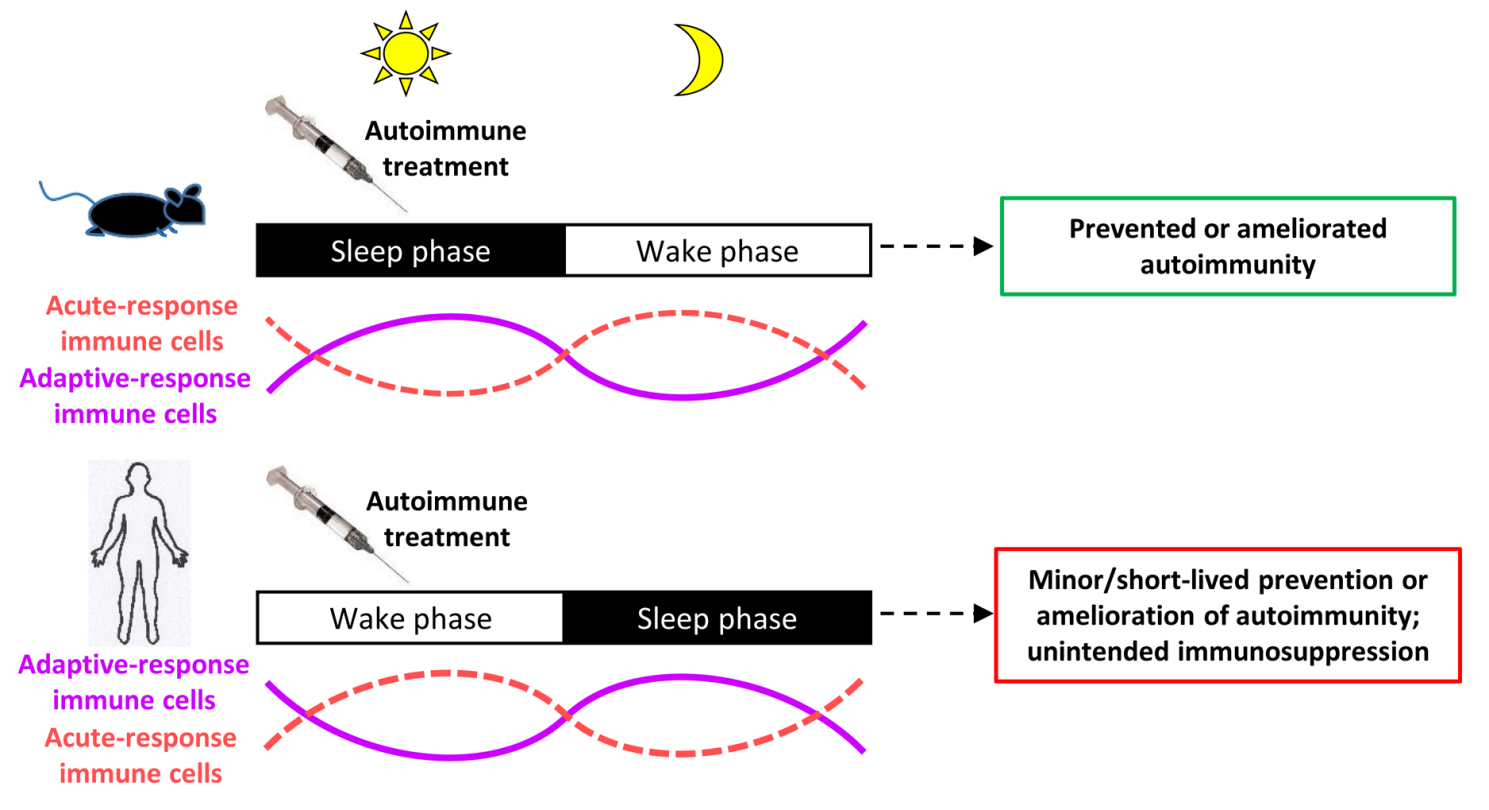
Autoimmunity treatments are applied at opposite immune system peaks in mice compared to humans. Humans and mice have different wake/activity and sleep/rest phases with regard to the daylight cycle. Because of this, researchers perform autoimmunity treatments at very different immune system peak times in humans compared to mice, which may account for some disappointing treatment trial outcomes in human autoimmunity.

Humans and mice have an opposite sleep/rest and wake/activity schedule in regards to the daylight cycle, but have the same oscillations in the innate and adaptive immune system in regards to the sleep/rest and wake/activity phases ^13^. Researchers tend to study mice primarily during the murine sleep/rest cycle, as that happens when the researcher is awake. Therefore, test treatments in mice affect immunological processes that are prevalent during the sleep/rest cycle, i.e. pro-inflammatory activities and memory cell formation ^13^. When translating the findings to humans, however, the opposite occurs. Human subjects are treated primarily during the wake/activity cycle, again for convenience. Therefore, patients are treated when anti-inflammatory mediators and local cytotoxic immune responses are active in the circulation but not when adaptive responses are prevalent. Since pro-inflammatory activities drive autoimmunity, and that type of activity occurs in the circulation mostly during the sleep/rest phase, the best window for treatment in humans may very well be missed ( Figure 1). Depending on the stability of a therapeutic, much of it may have been degraded or metabolized by the time the actual intended target is present in the circulation. In the case of therapeutic antibodies, which are generally considered to have good stability, the antibodies may be engulfed through endocytosis or pinocytosis ^28^ by cells other than the target cells and therefore become less available by the time the target cells are present. In addition, depending on what the particular therapeutic target molecule is, cells other than the intended ones, which also express that particular molecule and are present in the circulation during the wake/activity phase, may be targeted thus causing a broader immune suppression than intended.

One example of a therapeutic treatment that has a target shared by several different cell types is Anakinra. Anakinra is a recombinant form of the naturally occurring IL-1 receptor antagonist protein that binds IL-1 type I receptor (IL-1R1) ^29^. Several cell types express IL-1R1, including T, B, endothelial, microglia, hepatocytes, and synovial fibroblast cells ^30– 34^. When Anakinra binds IL-1R1, the binding of IL1-α and/or IL-1β is hindered and therefore any disease that is driven by those cytokines would theoretically be ameliorated by this treatment. Anakinra has demonstrated great success in some autoimmune diseases, for example Systemic Juvenile Idiopathic Arthritis (SJIA) ^29^, but in others, for example Type 1 Diabetes (T1D), there has been no clinical benefit of this therapy ^35^. It is possible that the reason for these vastly different outcomes is that, in SJIA, there is IL1-R1 expressed on synovial fibroblasts that are involved in the immune attack on the synovium and therefore Anakinra correctly targets the culprits and has a direct effect on the symptoms. In T1D, however, it is possible that IL1-R1 signaling is not directly responsible for symptoms; rather IL1-R1 signaling may bolster T and B cells that are the overall drivers of the disease. If that is the case, administering Anakinra during the wake phase will target cells that express IL1-R1 but are not directly involved in the disease process and by the time T and B cells are circulating, the Anankinra is no longer available.

An example of a therapeutic treatment that has a much more narrow target in terms of cell type is Rituximab. Rituximab is an antibody to CD20, which is expressed almost exclusively on B cells. Rituximab has demonstrated success in Rheumatoid Arthritis and Relapsing-Remitting Multiple Sclerosis but has shown no efficacy, or only transient efficacy, in for example Systemic Lupus Erythematosus and T1D ^36– 38^. It is possible that some autoimmune diseases have a more prominent role of B cells as causative cells and others do not and that could account for the different outcomes. However, it was shown that Rituximab caused reduced B cell numbers primarily in peripheral blood and less so in secondary lymphoid tissue ^36^. Interestingly, it was demonstrated that different subsets of B cells are more or less sensitive to anti-CD20 treatment and that if they were mobilized into the vascular space it rendered them more sensitive to anti-CD20 depletion ^39^. Conversely, if B cell egress from lymph nodes was prevented, the cells were not readily depleted with anti-CD20 treatment ^39^. This scenario is reminiscent of the circadian oscillations of B cells, entering and leaving the circulation/lymph nodes. Therefore, it is possible that, in specific autoimmune diseases, it will be important to administer the Rituximab when B cells are most prominent in the circulation in order to most efficiently deplete those cells.

## Timing and dosing in relation to toxicities and success of autoimmunity treatments

Recently it has become more apparent that there is a biologically and medically relevant impact of time-of-day in administering pharmaceuticals or in encountering environmental toxicants, where the time-of-day significantly modulates the efficacy and toxicity of the administered drug or encountered toxicant ^40^. The timing and dose of therapeutics administration in autoimmunity may also be tied to toxicities or immunosuppression. Often the thinking is that if a therapeutic with a reasonably long half-life is given in a high (but tolerated) dose then there will be a maximum effect for the patient. However, it is possible that by doing so, the therapeutic ends up targeting a much broader range of immune cells that may share expression of the same target molecule as the intended target cells, causing unnecessary immune suppression. For example, if a large dose of a T cell inhibitory antibody (targeting a molecule expressed on most T cells) is given during the wake/activity phase, it will first target CD8 effector and natural killer T cells since they are present in the circulation at that time. Subsequently, it will target the intended memory and naïve T cells once the sleep/rest phase is entered. This could happen several times over several days or weeks until the antibody is no longer available and may unnecessarily render the patient unable to respond to acute pathogen exposure. If the antibody instead is given in a smaller dose during the sleep/rest phase, it may adequately target the intended cells (memory and naïve T cells) but not be available to target other cells (CD8 effector and natural killer T cells) once the wake/activity phase is entered, thereby allowing for adequate acute pathogen response.

In the case of attempts to tolerize autoreactive T cells to self-antigens ^41– 44^, a similar scenario may be at play. Restoring tolerance with small doses of antigen works well in the setting of IgE driven allergy ^45, 46^. However, in autoimmunity there has thus far been very limited success ^8^. One hypothesis could be that, in allergies, the target cells/molecules that initiate the allergic response are those that are active during the wake/activity phase, which is also the period that human subjects normally encounter allergens. Therefore, administering a small dose of antigen during the wake/activity phase works to target the intended immune cells and is then, because of the low dose, no longer available by the time the sleep/rest phase commences. In autoimmunity, however, the intended target cells, B cells, CD4 helper T cells, etc. are more prominent during the sleep/rest phase. Therefore, administering the antigen to humans during the daytime may target the wrong cells and the small dose of antigen will be more or less exhausted by the entry into sleep/rest phase, resulting in inadequate targeting of the intended cells.

## Concluding remarks

Obviously, the circadian rhythm of the immune system may not be at play in the efficacy of all autoimmunity treatments. There are many other facets such as route of administration and efficacy of tissue distribution of any given therapeutic to take into account. However, considering the oscillations of immune cells in the circulation in some autoimmunity therapies may be prudent. Determining when the target molecules/immune cells are present in the circulation and administering small doses of the therapeutic at that point may maximize the effect and lessen unintended consequences. Currently, researchers are serendipitously targeting the culprit cells/molecules in autoimmune mouse model studies because of the difference in mice’s sleep/wake phases and ours. While it may be difficult to treat human autoimmunity during the sleep/rest phase, it may pay off with an increase in the therapeutic effect. To address this question, experiments can be done on autoimmune mice housed in an altered light cycle such that their sleep/wake cycle coincides with ours and therefore treatments can easily be applied when anti-inflammatory mediators and local cytotoxic immune responses are active in their circulation. Thus it can be ascertained whether the efficacy of the treatments lessen when applied during the wake/activity phase. While it may be more difficult to accomplish, in human trials of autoimmunity treatments it would be useful to assign some subjects to groups receiving treatment at different times during a 24-hour period, especially during the sleep/rest phase. Certainly, it would be worthwhile to revisit treatments that previously demonstrated great efficacy in mouse models but only slight improvements in translational treatments.

## Data availability

No data are associated with this article.
