# Supplementary material for: Are we aiming to miss in translational autoimmunity treatments?
Source: F1000Res. 2019 Jan 8;7:1754. Originally published 2018 Nov 6. [Version 2] doi: 10.12688/f1000research.16894.2 (PMC6259590; doi:10.12688/f1000research.16894.2)
Supplement: Peer review history

# Peer review: Referee response for version 1

**DOI:** 10.5256/f1000research.18471.r40304

**Published:** 2018-12-07

**Authors:** Jon Piganelli

**Affiliations:** 1 Department of Surgery, University of Pittsburgh School of Medicine, Pittsburgh, PA, USA

Vaitaitis and Wagner present an interesting hypothesis regarding the inability of effective immunotherapies for autoimmunity in mice to translate to human clinic. They describe the difference in sleep/wake cycle as a confounding factor in the translation. Where in mice, the majority of work is conducted during the rodent sleep cycle where as the authors state there is an increase in adaptive immune cells, those which are often targeted by these types of therapies. However these same treatments are given in the daylight/wake period of humans, where the adaptive immune system is less active. They propose to give these types of treatment during the sleep cycle in humans to facilitate the optimal targeting of the adaptive immune response to down-modulate the ensuing self-reactive response.

 Although this makes sense there are a few caveats that must also be considered. For example, the administration route and dose may have a profound impact on the overall immune response. Also for dosing and and timing we assume that tissue distribution is limited if at all, and that the agent is not getting to the target tissue. This is likely not the case, as then many agents would fail to have any real therapeutic value at all. I understand the logic but as we know pharmacokinetics is extremely complex for drug delivery. Notwithstanding, the complexity of this is far greater still, since the interaction of the circulating immune cells, for example CD4-helper cells must interact with APC/DC in the draining lymph node, where the APC has ferried antigen where it can present to T cells, This interaction then puts the APC at the center of the issue and therefore the whereabouts of these cells at the time of antigen exposure becomes critical.

 Overall, this article brings us out of the dark and into the light for just how much we take for granted in these types of experiments when moving to the clinic. Overall the author’s suggestions to assess these differences warrant that these types of experiments are investigated.  For example, the design of sustained delivery of agents that can be given in more conducive time frames that then release their payload during the sleep cycle may be impactful.

I have read this submission. I believe that I have an appropriate level of expertise to confirm that it is of an acceptable scientific standard.

# Response

**Published:** 2018-12-07

**Authors:** Gisela Vaitaitis

**Affiliations:** University of Colorado, Anschutz Medical Campus, USA

Dr. Piganelli,

 Thank you very much for reviewing our manuscript. We greatly appreciate your time. Your comments and insights are well taken and we agree that there are many levels of complexity in autoimmunity treatments. We also agree that the explanation proposed in our manuscript for the discrepancy in success of autoimmunity treatments between mice and humans does not necessarily fit all situations. Nonetheless, adjusting the timing of administration of treatments may be very important for some therapeutics with resulting increase in success.

# Peer review: Referee response for version 1

**DOI:** 10.5256/f1000research.18471.r40306

**Published:** 2018-11-22

**Authors:** Hubert M. Tse

**Affiliations:** 1 Comprehensive Diabetes Center, Department of Microbiology, School of Medicine, University of Alabama at Birmingham, Birmingham, AL, USA

Vaitaitis and Wagner have written an interesting opinion article regarding the shortcomings of mouse models of autoimmunity and their assessment of immunotherapies to delay disease progression in human clinical trials. Mouse models are instrumental in increasing our understanding of genetics and immune responses involved in disease pathogenesis. However, the identification of numerous interventions that can delay autoimmunity in mice are ineffective in humans. One potential scenario of the lack of success in translational studies may be due to opposite circadian clocks and sleep/wake phases in mice and humans. The authors provide an interesting opinion that is overlooked by immunologists and warrants attention in future studies.  The opinion article is well written, but could be strengthened by providing examples of how some immunotherapies including anakinra and rituximab have shown to be efficacious in mice, but not in human clinical trials with respect to opposite circadian clocks.

I have read this submission. I believe that I have an appropriate level of expertise to confirm that it is of an acceptable scientific standard.

# Response

**Published:** 2018-11-24

**Authors:** Gisela Vaitaitis

**Affiliations:** University of Colorado, Anschutz Medical Campus, USA

Dr. Tse,

 Thank you very much for reviewing our article. We are grateful for your time, input, and comments. We will take your comments into account when revising the manuscript to create version 2.

# Peer review: Referee response for version 1

**DOI:** 10.5256/f1000research.18471.r40305

**Published:** 2018-11-20

**Authors:** Li Wen

**Affiliations:** 1 Section of Endocrinology, Yale School of Medicine, New Haven, Connecticut, USA

Vaitaitis and Wagner published this timely opinion article regarding an obvious overlook of the circadian control of immune cells and the unsatisfactory outcome of clinical trials.  The authors rightly pointed out that most, if not all, immune-therapies for human autoimmune diseases have been based on animal model, especially mouse model, studies.  The mouse has a completely opposite circadian rhythm to humans.  This could be one of the reasons that many clinical trials of immune therapy cannot be faithfully translated to humans.  Pre-clinical studies, if not specific for circadian research, are all carried out in the daytime, when the mouse normally sleeps, whereas clinical studies in humans are conducted in the daytime, which is the human waking phase.  In addition to the difference in circadian regulation of immune cell functions, importantly, the circadian regulation of metabolism of immune cells needs to be taken into consideration in the interpretation of the “failed” immunotherapies in humans, which have had positive outcomes in mouse model studies.  The authors have made a very important point in highlighting this major overlooked possibility for some of the discrepancies in efficacy of treating autoimmune diseases, such as type 1 diabetes, between the mouse and human studies.

I have read this submission. I believe that I have an appropriate level of expertise to confirm that it is of an acceptable scientific standard.

# Response

**Published:** 2018-11-20

**Authors:** Gisela Vaitaitis

**Affiliations:** University of Colorado, Anschutz Medical Campus, USA

Dr. Wen,

 Thank you very much for taking the time to review our manuscript. We greatly appreciate your time, interest and comments.

# Peer review: Referee response for version 1

**DOI:** 10.5256/f1000research.18471.r40307

**Published:** 2018-11-12

**Authors:** Chris Kevil

**Affiliations:** 1 Department of Pathology, Louisiana State University Health Shreveport, Shreveport, LA, USA

This is an opinion article addressing the question whether missed opportunities have occurred due to inconsistent immune intervention studies between mice and men involving differential time of intervention.  The proposed hypothesis is interesting and potentially plausible given the recent insights into circadian regulation of immune responses.  However, the current opinion piece is not fully substantiated in certain areas requiring revision.

 Are arguments adequately supported by evidence from the literature?

 While the authors cite some reports highlighting circadian regulation of immunity, insufficient discussion was provided regarding specific differential immune responses and how they may actually impact tolerance strategies.  Further discussion of this area would strengthen the work.

I have read this submission. I believe that I have an appropriate level of expertise to confirm that it is of an acceptable scientific standard, however I have significant reservations, as outlined above.

# Response

**Published:** 2018-11-16

**Authors:** Gisela Vaitaitis

**Affiliations:** University of Colorado, Anschutz Medical Campus, USA

Dr. Kevil,

 Thank you very much for taking the time to review our paper. We greatly appreciate your input and agree that we neglected to discuss the specific immune responses that are relevant in autoimmunity. We will update the manuscript to reflect this once we have the comments from other reviewers as well.
